# 2D dynamical arrest transition in a mixed nanoparticle-phospholipid layer studied in real and momentum spaces

**DOI:** 10.1038/srep17930

**Published:** 2015-12-10

**Authors:** Davide Orsi, Eduardo Guzmán, Libero Liggieri, Francesca Ravera, Beatrice Ruta, Yuriy Chushkin, Tiziano Rimoldi, Luigi Cristofolini

**Affiliations:** 1 Dipartimento di Fisica e Scienze della Terra, Università degli Studi di Parma, Parma, Italy; 2 Consiglio Nazionale delle Ricerche - Istituto per l’Energetica e le Interfasi, U.O.S. Genova (CNR IENI), Genova (Italy); 3 ESRF- The European Synchrotron, CS 40220, 38043 Grenoble Cedex 9, France

## Abstract

We investigate the interfacial dynamics of a 2D self-organized mixed layer made of silica nanoparticles interacting with phospholipid (DPPC) monolayers at the air/water interface. This system has biological relevance, allowing investigation of toxicological effects of nanoparticles on model membranes and lung surfactants. It might also provide bio-inspired technological solutions, exploiting the self-organization of DPPC to produce a non-trivial 2D structuration of nanoparticles. The characterization of interfacial dynamics yields information on the effects of NPs on the mechanical properties, important to improve performances of systems such as colloidosomes, foams, creams. For this, we combine micro-tracking in real-space with measurement in momentum-space via x-ray photon-correlation spectroscopy and Digital Fourier Microscopy. Using these complementary techniques, we extend the spatial range of investigation beyond the limits of each one. We find a dynamical transition from Brownian diffusion to an arrested state driven by compression, characterized by intermittent rearrangements, compatible with a repulsive glass phase. The rearrangement and relaxation of the monolayer structure results dramatically hindered by the presence of NPs, which is relevant to explain some the mechanical features observed for the dynamic surface pressure response of these systems and which can be relevant for the respiratory physiology and for future drug-delivery composite systems.

Self-assembly and self-organization of nanoparticles (NP)–surfactant mixed layers at the liquid-liquid or air-liquid interface are exploited in biomedicine, materials science, food science or renewable energy^1^,^2^. Examples of these applications are colloidosomes^3^, obtained from nanoparticles self-assembled at the droplet interface in emulsions^4^, which present a large potential for applications in the controlled release of active agents (drugs, pesticides, self-healing agents, flavors, nutriceuticals, etc.). Particle-surfactant self-assembly is also exploited in microfabrication^5^,^6^ and for the preparation of 2D functional materials for optoelectronics^7^ and membranes with antibacterial activity^8^. Less ordered systems are instead often concerned with emulsions and foam stabilization^9^ and are used in the development of porous materials^10^. In all soft systems, the study of spontaneous fluctuations and of the related mechanical response takes on a practical relevance in relation to the processing, to the durability and the shelf-life. Just to mention a relevant example, local dynamics residually found into the deep frozen state of a glass affects drug stability and availability^11^,^12^. On the other hand, spontaneous fluctuations and rearrangements, if properly controlled, may be used to generate desirable properties –as in the case of the solidification of a colloid-stabilized cream under gravity, yielding a compact non-crystalline layer^13^. These fluctuations are also relevant in some biological interfacial processes, such as in the case of NP–phospholipid mixed layers, which, besides being inspirational for future hybrid bio-inorganic applications^14^,^15^,^16^, are also useful to investigate toxicological issues and to understand the adverse effects of particles on the respiratory physiology^17^. The most important physiological role during the respiratory cycle seems to be played by the rearrangement of the pulmonary surfactant layer structure, which, in fact, warrants the proper variation of the mechanical properties of alveoli^18^. These properties are affected by the incorporation of NPs. In particular, significant changes of the dilational elasticity pattern have been observed^19^, as well as an important increase of the non-linearity of the response of the surface tension to sinusoidal area variations under conditions simulating the respiratory cycles^20^. The latter indicates how NPs interfere with the process of respreading of the pulmonary surfactant during the alveoli expansion.

Our work focuses therefore on the study of the microscopic spontaneous fluctuations of a model system, employing a combination of scattering and microscopy techniques to cover a broad spatial and temporal region (Fig. 1). The system is depicted in Fig. 2, and is formed at the air-water interface by a mixed layer of silica nanoparticles and DPPC (1,2-dipalmitoyl-sn-glycero-3-phosphocholine) - the most abundant phospholipid in human lung surfactant (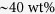) and also common in cell membranes in general. We present a characterization of its spontaneous 2D dynamics within the spatial scale range of about 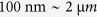 and within 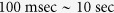 on the time scale, in different conditions of surface density/pressure. We find a transition between different dynamical regimes, all depicted in Fig. 2, on increasing the surface pressure, which leads to intermittent collective rearrangements in the arrested state.

In multicomponent systems such as that studied in this work, the complexity of the interfacial dynamics results from the superposition and coupling of different dynamic processes. The very appearance, time-scales and peculiarities of these dynamic processes are strictly dependent on the morphology and structure. To tackle this complexity, different approaches have been proposed, including mechanical and optical techniques. A very recent study of the coarsening dynamic of a colloidal gel’s spinodal decomposition^21^ combined direct-space particle tracking data with Q-space data generated by Fourier-transform of real space imaging data, thus elucidating anomalous, superdiffusive dynamics, presumably related to residual stresses in the material upon coarsening of a phase separated structure. On the contrary, in the present work we combine data measured *directly* in Q-space by a scattering technique, namely Grazing Incidence X-ray Photon Correlation Spectroscopy (GI-XPCS), with real-space imaging data.

## State of the art

### NP-DPPC mixed layers at the air/water interface

The phase behavior of DPPC at the air/water interface has been widely investigated also for its relevance to the understanding of the mechanical behavior of alveoli during the respiratory cycle^22^. In addition to the important dynamic effects mentioned above, the incorporation of silica nanoparticles in the monolayer -either from the subphase or co-spread- induces significant modifications in the surface pressure-area isotherm of DPPC monolayers. In particular, the coexistence region between liquid compressed (LC) and liquid expanded (LE) phases disappears^23^,^24^,^25^, the isotherm shifts to higher areas per molecule and the collapse pressure is reduced. The quasi-static surface elasticity in the condensed states is also reduced^20^,^23^. These observations call for a decrease of the interaction between the phospholipids’ molecules in these mixed monolayers. Direct information on the above structural modifications has been obtained in a previous study^26^ based on fluorescence microscopy. We have shown that when NP are incorporated into the surface, upon compression, a mixed NP/DPPC phase if formed, which coexists with disks formed by pure LC domains of DPPC. Fluorescently labeled lipid molecules are confined in the mixed phase, therefore, pure DPPC regions appear black in epifluorescence imaging. Even at the highest attainable pressures, the film morphology is characterized by these black, round features, which consist of pure DPPC, and are due to the LC domains, which resist to the penetration/diffusion of NPs or fluorophore molecules. At high surface pressure, these disks form a densely packed 2D structure. The mechanical moduli of this mixed monolayer are somewhat smaller than those of the pure DPPC monolayer at the same pressure.

### Arrested states

**Figure 2:**
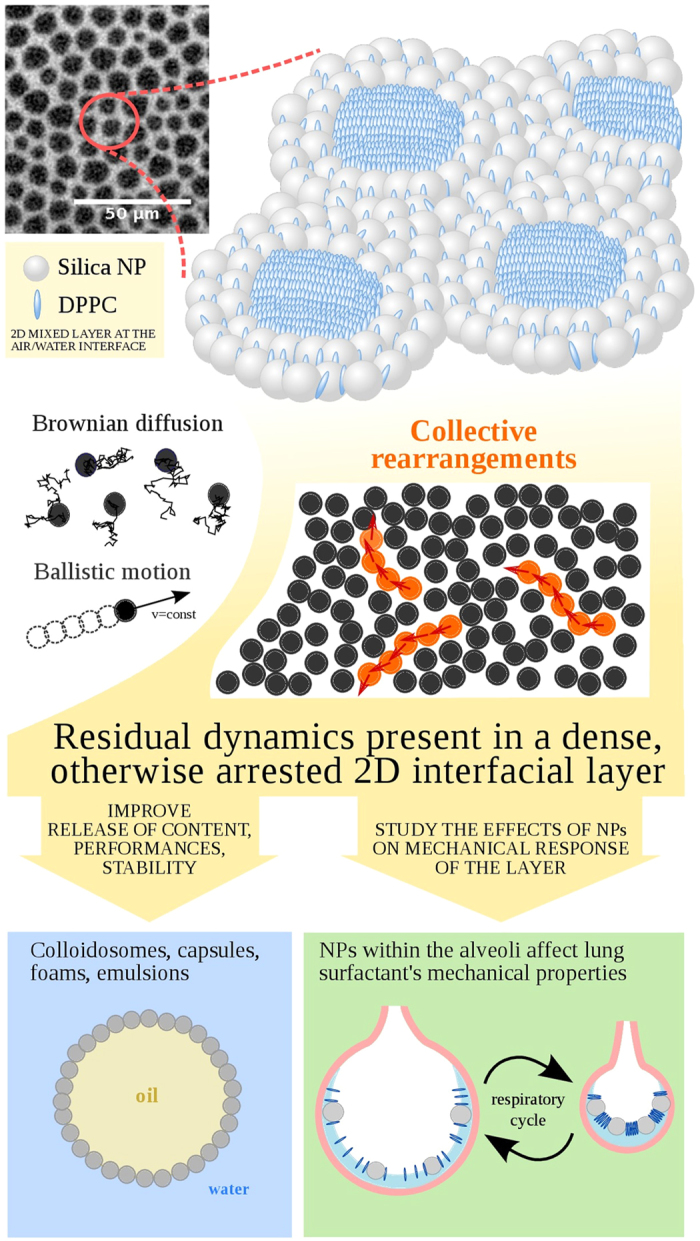
From top to bottom; schematization of the interfacial system whose dynamics is investigated in this work (elements not to scale). We characterized its microscopic dynamics as a function of surface concentration; we discriminate between different dynamical regimes: from the simple Brownian diffusion and Ballistic motion to Collective Rearrangements, similar to what is found e.g. in traffic jams. The outcome of this study might impact on technological applications such as colloidosomes, capsules, foams and emulsions. Moreover, it might lead to a better understanding of the effect of nanoparticles on lung surfactants and cell membranes in general.

Despite their apparent distinct nature, colloidal suspensions, gels, metallic glasses, fluid lubricants, pharmaceutical compounds, window glasses, granular media and emulsions owe their properties to the fact that they can be driven into an out-of-equilibrium configuration, in which they cease to flow^27^. Detailed information on the dynamics of many of these systems can be obtained by techniques such as XPCS, its visible-light counterpart Dynamic Light Scattering (DLS) and Digital Fourier Microscopy (DFM). All these techniques operate in the momentum space, or Q-space 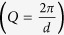, where *d* is distance in real space. However, experimental results have to be interpreted through a model to yield significant quantities, and the choice of the model is a crucial point. Some of the most relevant dynamic regimes are sketched in Fig. 2: In the simple case of Brownian diffusion^28^, an intermediate scattering function (correlation function) has a simple exponential decay with characteristic time 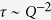. In this scenario macroscopic mechanical properties (e.g. viscosity) relates with the observed microscopic fluctuation dynamics (e.g. diffusion coefficient) via Stokes-Einstein relations. However, this simple picture breaks in arrested states. For instance, in the case of polymeric layers or 2D gel networks in which the correlation functions have compressed exponential form^29^,^30^ with characteristic time 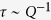, defining diffusion coefficients is not trivial. Scenarios that are even more exotic have been observed e.g. in aged colloidal creams^13^, in aged Laponite^31^ and in methylcellulose^32^ gels, systems where stretched exponential correlation functions are found with 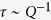. Many of these dynamical features are interpreted postulating either discrete intermittent rearrangements or random relaxations of stress fields, whose direct observation in real systems has been so far rather elusive^33^. Of course, the uncertainty about the origin of the different 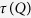 dependences poses tremendous problems when trying to relate the microscopic fluctuation dynamics with macroscopic mechanical measurements, where the spatial scale probed is so large that, in Q-space, they correspond to the macroscopic limit 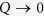. On the other hand, other techniques such as Multiple Particle Tracking (MPT) directly investigate the dynamics, with the advantage of being able to indicate the most appropriate model. However, this technique can only be used on objects that can be seen under the optical/fluorescent microscope. Moreover, it often suffers from limited statistics, due to the small area that can be viewed and tracked simultaneously under the microscope objective. In comparison, scattering techniques such as XPCS access the dynamics on similar temporal windows, but on slightly different spatial scales. Consequently, there is a significant overlap region between the two, allowing for a validation of the results through detailed comparison. At the same time, the combination of these two techniques spans a much wider spatial range than any single technique.

## Results and Discussion

**Figure 3:**
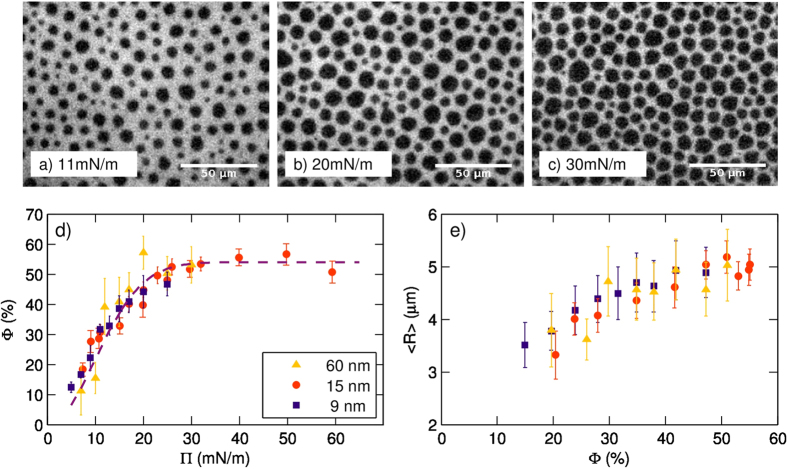
Silica NPs suppress domain coalescence, stabilizing a gel-like structure for the DPPC film and giving domains a regular, roughly circular shape. (**a–c**) Epifluorescence images taken at 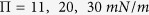 for silica particles of size 60 nm. (**d**) The fraction of interface 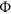 covered by the disks is reported as a function of surface pressure 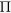 for silica particles of different radii; after a steep rise up to 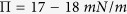 and 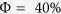 more week dependence is found at higher pressures. a saturation value is reached for 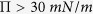. The dashed line is a guide to the eye; error bars report standard deviation over several (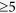) images of different regions. (**e**) Mean radius *R* of the disks, measured as a function of 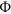 for different values of the NP sizes. Error bars report standard deviations over several observations.

Epifluorescence microscopy was used to follow the morphological changes in the film during compression. Figure 3 reports the morphology of the film -containing 60 nm silica particles- at different compression levels: the images shown here have been taken at a surface pressure 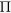 of 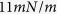 (panel a), 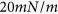 (b) and 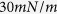 (c), above the pressure of the LE-LC coexistence plateau in pristine DPPC. The most relevant change observed is the increase of the fraction of surface occupied by regions of densely packed DPPC molecules -which appear black because all the fluorescent molecules have been expelled. The fraction 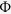 of air/water interface covered by them has been measured by a judicious choice of a threshold value the epifluorescence images; as 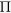 is increased, it increases up to 54% (panel d); this saturation value is reached at 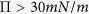. On panel e of the same Figure, we report the average radius 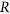 of these disks as a function of 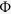: in the explored range, the change of *R* is limited and it saturates at 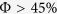.

Such situation is thus drastically different from pristine DPPC monolayers, since the presence of NPs hinders the formation of a continuous layer of the DPPC LC phase.

The results reported in Fig. 3 are consistently reproducible and it is worth noticing that the observed behavior is practically independent from the particle size, for diameters within the 10–100 nm decade. Therefore, we are able to exploit the one-to-one correspondence between 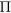 and 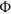 (through the 2D geometry provided by Langmuir layers) to assign a specific structural state to the system in correspondence of specific values of 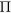. This is in particular important during the XPCS measurements, when direct microscopy imaging is unfeasible.

### Dynamics in real space

A direct approach to the measurement of the dynamics is to track in time the position of the black, disks that characterize the film morphology. This approach is strictly analogous to the more common Multiple Particle Tracking (MPT) technique^34^; we opt for a different name to stress that we are not tracking the displacements of individual silica nanoparticles but that of the remnants of DPPC domains. Their nearly circular shape simplifies the measurement of their position in epifluorescence images; we follow their displacement in time by recording videos and performing accurate position tracking.

### Microscopic Tracking: From a Brownian regime to an arrested state

**Figure 4:**
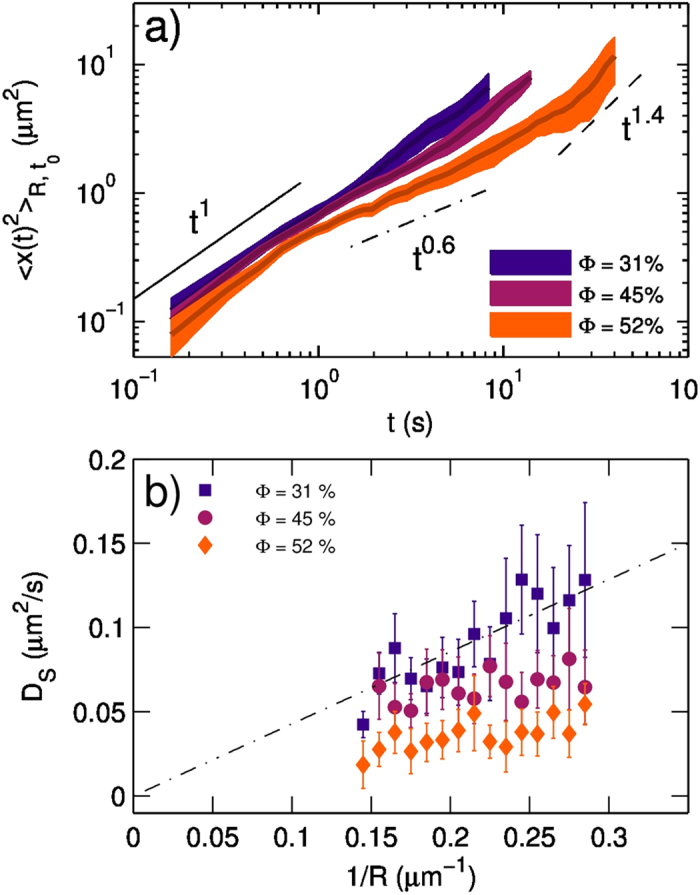
At low 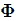, Brownian diffusion of the domains in the Hughes regime is observed and the coefficient 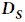 scales with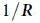, as expected in Hughes model. At higher 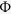, 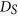 becomes R-independent, a sub-diffusive trend emerges at intermediate time, followed by a super-diffusive regime. This indicates a transition to an arrested state. (**a**) 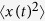 grows linearly in time at 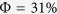, indicating diffusive dynamics (black solid line). At higher concentration(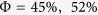) the linear trend holds up to 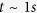. For 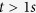 we find a sub-diffusive regime (m < 1, black dash-dot line), followed by a super-diffusive trend (m > 1, black dashed line). These values for 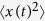 come from an average on starting time *t*_0_ and on all the domains having the same radius (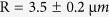) in the field of view; color regions indicate standard deviations calculated over the ensemble. (**b**) Average values for the diffusion coefficient *D*_*S*_ (describing the diffusion regime found at 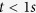) are reported as a function of 1/R. Error bars are standard deviation. At 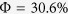 we find 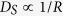, in agreement with Hughes model, (eq. (3), dotted line), while at higher values of Φ this relation breaks.

From the trajectories measured at different values of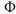, we calculate the mean square displacement 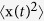 as a function of time; results are reported in Fig. 4a -on a double-logarithmic scale- for disks with a comparable radius (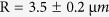) and at three different values of surface concentration (namely, 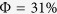, 45%, 52%). As Supplementary Information, in Figure S1 we also report 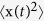 for different values of *R*.

A linear growth of 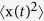, as would be expected in the case of Brownian diffusion, is indicated by the continuous black line. At low surface concentration (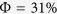), 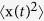 follows this trend in the full temporal range explored. On the contrary, as concentration is increased, the situation becomes more intriguing as different regimes are found. For instance, at 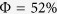, at early times (below 1 sec- indicated by the vertical red halo in the Figure) 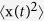 still grows linearly. In this regime, a “short time diffusion coefficient” *D*_*S*_ can be defined as


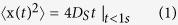


At intermediate times (approximately 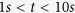) the growth becomes sub-linear, indicating that the diffusion is hindered by the presence of adjacent objects. Finally, at longer times (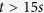) the tracked objects overcome this hindrance, a diffusive or even super-diffusive regime is found. All this can be quantified by a power law


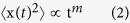


In which the exponent *m* quantifies the linear (*m* = 1), sub-linear (*m* < 1) and super-diffusive (*m* > 1) trends respectively.

Diffusive regimes of large flat inclusions in an interfacial film have been studied theoretically long ago by Hughes and coworkers^35^. Their results were verified experimentally for DPPC monolayers by Klingler and Mc Connell^36^. This regime holds when the diffusing object has size R much larger than the so called Saffman-Delbruck length, given by 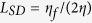, where 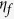 is the 2D viscosity of the film, while 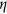 is that of the bulk phase. In this regime, diffusion is limited by the drag exerted by the subphase and not by the surrounding film. Then, the diffusion coefficient D is given by:


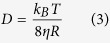


Where T is the temperature (293 K in the present case) and 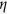 is the subphase viscosity, (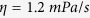, for the NP subphase, a value slightly larger than that of pure water at this temperature).

In Fig. 4b we report the mean values of *D*_*S*_ as a function of the inverse of the radius 1/R, for different values of 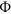. The linear growth of eq. 3, indicated by the dashed line, is expected whenever the Hughes regime is verified. This is the case for the data at 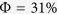. On the contrary, at higher 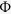, the diffusion coefficient becomes less dependent, or even not-dependent, on the size R. In principle, this could be ascribed either to an arrest transition or to the transition from the Hughes regime to the Saffman-Delbruck^37^ regime, as the latter predicts a weak logarithmic dependence of the diffusion coefficient on the radius R. However, we rule out this latter hypothesis, as this transition occurs when 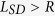, which cannot be our case as 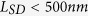 and 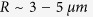. Therefore, the transition is from the Hughes diffusive regime to an arrested state.

### Residual dynamics in the arrested state

**Figure 5:**
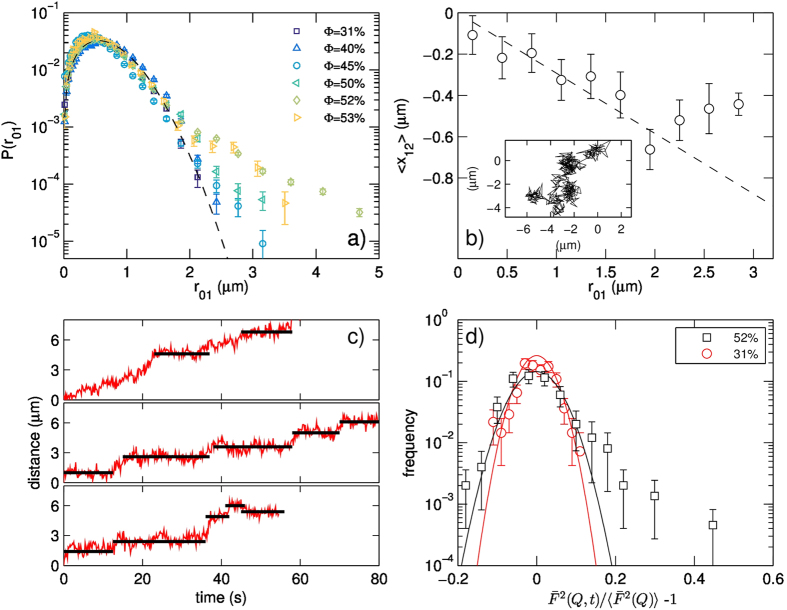
(**a**) Self part of the Van Hove Correlation functions of the trajectories reported in Fig. 2, calculated for 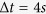, for increasing values of 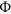. The values of 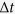 is chosen to correspond to the sub-diffusive trend of the mean square displacement found at high 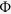. At 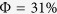 the data points lie on a Gaussian curve (dashed line), compatible with Brownian diffusion. At higher concentration, the distribution develops tails that are broader than in a Gaussian, indicating more frequent occurrence of long-range jumps. This is in agreement with the dynamical arrest picture. (**b**) The average value of the projection 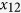 of a step 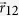 along the direction of the previous step 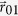 is reported as a function of the size 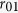 of the first step. Steps have been measured at 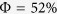 over time interval 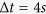. The data follows a linear decrease (dashed line) which implies anticorrelation between the first and second step: this happens when one of the tracked object hits the “cage” of the surrounding neighbors and is pushed back towards the previous position. A deviation from the linear trend is observed for 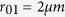; this length corresponds to the size of the “cage”. The inset shows a typical trajectory measured at 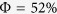; the trajectory presents clusters of positions that are roughly 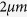 in size. (**c**) Displacements of three different objects measured at 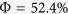, reported as a function of time. They present long periods of short range movements around a stationary position, followed by sudden jumps. Black lines highlight the time intervals between successive jumps. (**d**) Histogram of temporal intensity fluctuations of the Fourier transform of successive images for two values of 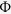 with respect to the time-averaged value, measured at 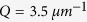. While at 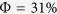 the fluctuation histogram is compatible with a Gaussian curve (solid red line), at 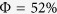 the distribution is characterized by a tail, which is broader than Gaussian, indicating that rearrangement events are taking place in the layer.

Additional insight into this arrested state is gained by computing the self-part of the van Hove correlation function of the displacements from the trajectories of the individual domains. Following the approach proposed by Doliwa and Heuer^38^, we select a time interval 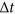 and calculate the displacements 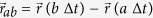; the self-part of the van Hove correlation function 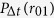 is defined as the probability distribution of 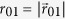. Figure 5a reports 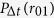 for different values of the surface concentration; the curves have been calculated at 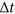 = 4 s, a time interval at which, at high surface concentration, we observe the sublinear power law trends of 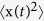.

At low surface concentration (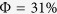) the disks undergo Brownian diffusion –a process known to yield a normal distribution- 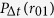 has a Gaussian shape (solid line). As 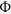 increases, we observe broader tails indicating the presence of long-range rearrangements; this imply that a non-negligible fraction of disks moves on large distances while the majority of them diffuse on short range, being limited by the presence of their neighbors.

The population of long-range moving objects can be indirectly quantified by means of the non-Gaussian parameter *α*_2_, which compares the fourth moment *μ*_4_ of the distribution *P*(*x*) of the individual components of the displacements, either *x* or *y*, to the second moment *μ*_2_^39^:


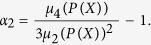


**Figure 6:**
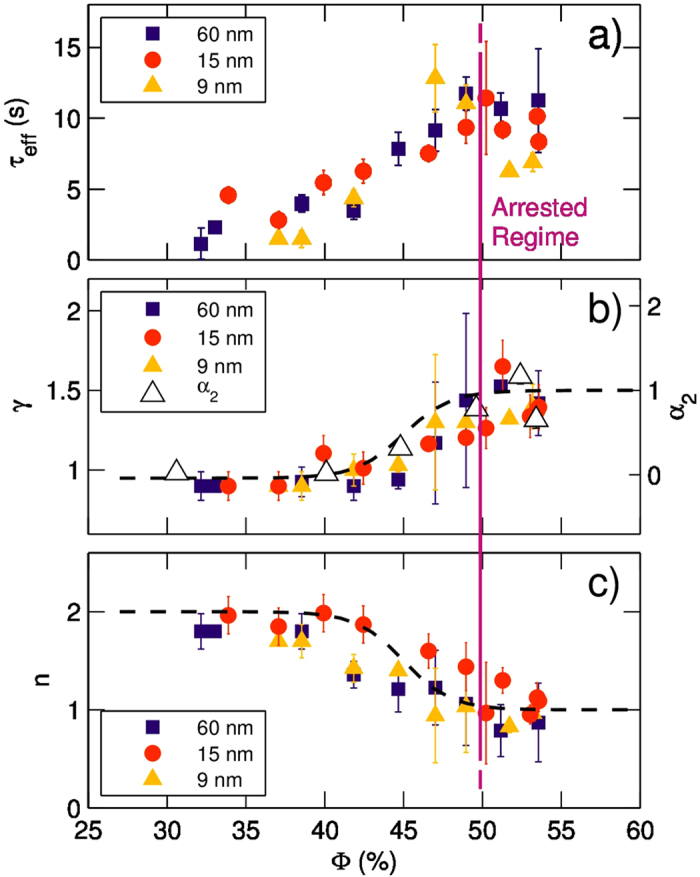
Diffusive dynamics is observed by XPCS at low concentration and up to 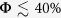. As 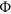 grows we find an intermediate regime of caged diffusion, with relaxation times growing with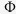. For 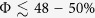 (vertical line) an arrested regime is reached, in which the only surviving motions are intermittent rearrangements.(**a**) On the left axis, we report 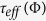 extracted from XPCS at 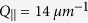; different symbols/colors indicate different NP sizes. As concentration increases, 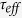 grows, marking the onset of dynamical arrest. (**b**) The shape parameter *γ* (right axis) increase from 1 up to 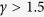 as 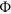 grows from roughly 40% –48−50%. In the same plot we also report the “non-Gaussian” parameter *α*_2_ which increases from 0 (Brownian case) to higher values. (**c**) In the same 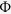 range, the 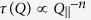 dependence switch from 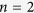 (diffusion) to 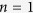 (ballistic-like). Overall, the trends of *γ*, 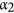 and 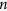 indicate the presence, in the arrested state, of sudden, intermittent rearrangements. Colors indicate different NP sizes.

This parameter is zero for a strictly Gaussian distribution and becomes larger when the tails of the distribution become more important than in the Gaussian case. It is reported in Fig. 6b: at 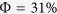 we found 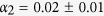, a value compatible with a purely Gaussian distribution; on the contrary, at increasing concentration, *α*_2_ increases, reaching 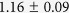 at 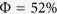, thus indicating at this concentration a strong deviation from the Brownian diffusion.

To verify if this deviation arises from caging effects, following the literature^40^ we calculated the anticorrelation between successive steps, quantified by the parameter


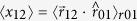


Which corresponds to the projection of 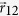 along the direction of 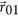, where 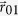 and 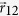 are two successive steps. In Fig. 5b we report this quantity as a function of the step length 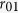 for the case at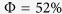. We find that it decreases with increasing values of 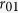 (dashed line), indicating the existence of an anticorrelation of 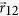 and 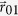 indicative of the presence of caging. However, when the first step is sufficiently large to exit the cage of the surrounding neighbors, the anticorrelation between subsequent steps is weakened; in the graph, this is indicated by a deviation from the linear trend indicated by the dashed line. In our system, this happens for 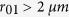. This value is considered to be an estimate of the size of the cage^40^. Inspection of a typical trajectory -reported in the inset of Fig. 5b- confirms that in the arrested state the disks rattle in “cages” about 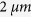 in size, with intermittent jumps over longer distances. Figure 5c reports the square displacements (i.e. not averaged neither over the statistic ensemble, nor over the initial time *t*_0_) for three different disks, measured at 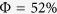; they shows the long periods of slow diffusion or vibrational motion around a static position, followed by sudden long jumps over distances of roughly 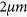.

The arrest transition, along with the hints of the presence of rearrangements found in the arrested state by means of the van Hove correlation functions, calls for a detailed investigation of localized dynamics, which somehow hits the limits of the tracking analysis performed so far. In order to gain a deeper insight, we apply a scattering technique, which holds the promise *i)* to extend the spatial range, and *ii)* to improve the statistics by measuring a larger portion of surface at the same time. In order to achieve this, in the following, we compare the results obtained in direct space with those found in the reciprocal space.

### Dynamics in momentum space

This motivates the investigation by Grazing Incidence X-ray Photon Correlation Spectroscopy (GI-XPCS), a scattering technique that allows a quantitative characterization of the interfacial dynamics^29^,^41^,^42^, providing insights into even shorter spatial scales and faster times (down to 10 ms) with respect to the ones accessed by direct-space imaging. The high statistics arising from data collection over the whole footprint of the x-ray beam, results in better signal-to-noise ratio in the analysis of tiny displacement if compared to the tracking experiments. The results can be *directly* compared with those from microscopy imaging by employing a novel technique named Digital Fourier Microscopy (DFM)^43^, also known as Digital Fourier Imaging^44^, which casts the information into reciprocal space by applying the Fourier transform to the epifluorescence images used for MT.

The basic quantity measured by XPCS (and DFM) is the autocorrelation function 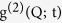 of the signal measured at a given scattering momentum Q. The decay of the intensity functions 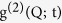 can often be described by the Kohlrausch-William-Watts expression,


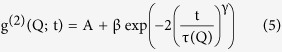


We track the evolution of the dynamics as a function of 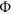 in terms of changes in the relevant parameters of the fit. In particular, we focus on the relaxation time 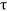 and on “fingerprints” of the dynamical character, namely the shape of the correlation function - represented by the exponent 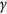
- and the dependence of the relaxation time from Q, given by the exponent n:


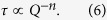


In combination, these parameters allow us to discriminate between different dynamical regimes within the limits cases of diffusive (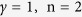) and ballistic (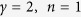) motions. To take into account the effects of possible changes of shape of the exponential decay, we express the relevant time scale of the dynamics using an effective relaxation time, which is calculated as the first moment of 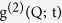 yielding


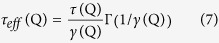


where 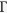 is the gamma function.

A peculiarity of the grazing-incidence scattering technique GI-XPCS with respect to microscopy is that it is not bound to 2D plane of the interface; by looking at the component of Q perpendicular to it, it is possible to measure the dynamics in the transverse direction. In our case, however, we find that the dynamic is confined at the air/water interface: its relevant parameters depend only on the component of the scattering vector parallel to the interface 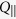, and displays no dependence on the perpendicular component 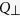 (more details are reported as Supplementary Information).

### Dynamical arrest and intermittent rearrangements

By means of GI-XPCS we could follow in detail the dynamical arrest transition. The growth of the relaxation times 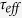 as a function of the concentration is reported in Fig. 6a. In correspondence to the slowing down of the dynamics, we also observe a change in the shape of the KWW decay^5^, as indicated by the exponent *γ* shown in 5b, and the coefficient *n* indicating the Q- dependence^6^, which is shown in 5c. Data points correspond to the average value obtained from four repetitions of the measurement in different spatial position of the interface; error bars are standard deviations.

Roughly speaking, three dynamical regimes are met with increasing concentration: up to 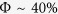 we find 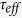 independent of 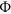. The dynamics is Brownian: correlation functions decay as simple exponentials (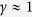) and the relaxation time depends on 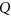 as 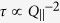. This framework nicely corresponds to what already indicated by the MT technique, namely *m* = 1 (linear increase in time of 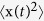) and *α*_2_ being very close to 0 (Gaussian distribution of displacements). Then, between 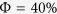 and 48–50% we observe a steady increase of 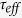 with 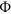. In this regime, the mechanical modulus of the film, as measured by the oscillating barrier techniques, increases as well, following the same trend of the XPCS relaxation time (more details in Fig. S6 of Supplementary Information) This is an example of the ubiquitous Stokes-Einstein generalized relations and is similar to what found in many other systems, including Langmuir monolayers of gold nanoparticles^45^ and nanoparticles in polymers^46^. Together with this steady increase of the relaxation time, we find evidence of a phase transition between two different dynamical states; we observe a continuous change of the shape of the correlation function and of the Q-dependence of the relaxation time, indicated respectively by *γ* and *n*. As the concentration is increased above 50%, the shape linearly increases towards faster-than-exponential decays (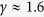) with 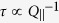, a dynamics commonly found in systems undergoing dynamical arrest^45^,^47^.

Analogous results are found by means of DFM: we analyzed in momentum space the epifluorescence images measured at 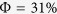 and 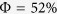. At lower value of 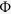 we find that 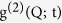 decay as a simple exponential (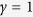) and 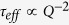, indicating Brownian diffusion –as expected by MT results. At 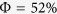, however, we find a compressed exponential shape (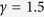) and a very weak dependence of 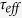 over Q.

The presence of intermittent rearrangements in the high-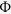 regime, evidenced in 4c, is quantified by the analysis of the temporal fluctuations of the Fourier transform intensity of difference of consecutive epifluorescence images, as in the similar technique Differential Dynamic Microscopy^21^. In Fig. 5d we report the histogram of the temporal intensity fluctuations of the signal, with respect to the time-averaged value, for two values of 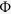; While at 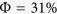 the fluctuation histogram is compatible with a Gaussian curve (solid red line), at 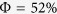 the distribution is characterized by broader tails, indicating large fluctuations due to the presence of sudden rearrangements in the layer. Within this respect, this results is complementary to the emergence of a non-Gaussian shape in the distribution of steps found by MT.

We have then a consistent picture in which the growth of relaxation times goes together with *i)* the increase of the non-Gaussian parameter 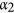 of the van Hove correlation functions, *ii)* the change in the shape of the relaxation, which turns from simple exponential (compatible with the Brownian case) to compressed shape, reported in Fig. 6b, *iii)* the Q dependence of 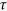, reported in 5c.

**Figure 7:**
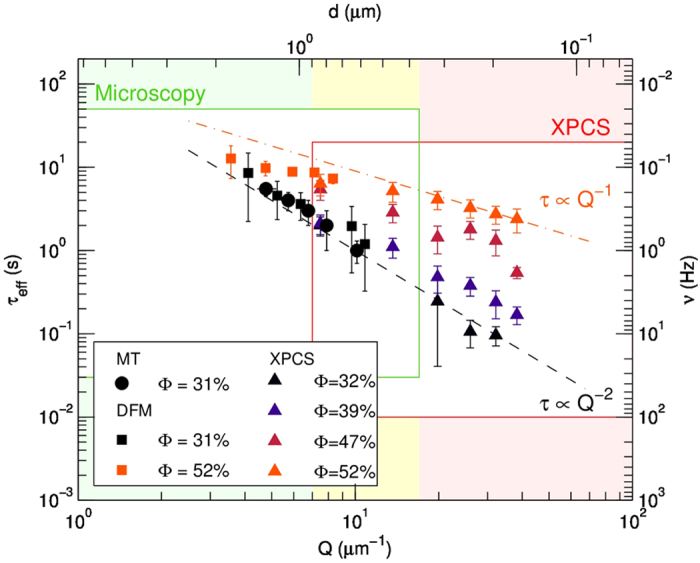
Comparison of the techniques: green and pink areas correspond to the domains of Microscopy and XPCS respectively; the framed region is covered by the present experiments, while generally accessible region is indicated by the larger shadowed area. The yellow area is the overlap. We report XPCS relaxation times 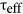 (colored triangle) and DFM times (squares) as a function of Q, and MT times as function of displacements (circles). The diffusive regime, 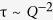 is the steeper dashed line, while the arrested regime, 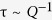 is the less steep dashed line. Correspondent frequencies 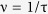 and distances 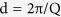 are reported on right and top axes for clarity. Note at each given concentration the good overlap of the results obtained by the different techniques.

Figure 7 summarizes all the results obtained by the different techniques operating in real and momentum space, by putting them on a log-log scale in which position and momentum (related by 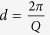) are on the horizontal axis, while times and frequencies 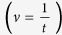
are in the vertical axis. Relaxation times obtained from MT are circles, XPCS are triangles of different color for different concentrations as indicated in the caption, and DFM are colored squares. The two colored areas correspond to the domains of the different techniques, whose overlap is marked in yellow. The combination of MT and GI-XPCS allow us to extend significantly the Q-range over which we are able to evaluate the 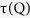 dependence: we find that the diffusive regime at low-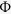 yields 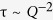 (steeper dashed line) while as concentration is increased, the transition to dynamical arrest is highlighted by the less steep Q-dependence of 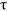, with 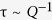 found at 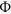 = 52% (less steep dashed line).

A compressed relaxation shape is predicted within a detailed model proposed by Bouchaud and Pitard^48^ for the dynamics in an elastic gel. Within this model, randomly appearing dipolar stresses generate a field of strains in the network of the elastic gel. It is then assumed that the dynamics of the diffusors is determined by the continuous relaxation of such local strains. As a function of the extension of the experimental time scale compared to the time of this relaxation process, different asymptotic time- and Q-dependences are predicted for the shape of the correlation function, which in our model correspond to the parameter *γ* varying from 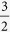 to 1. A slightly different phenomenological model was more recently put forward by Duri and Cipelletti for gel systems^49^ postulating the dynamics to be governed by rare, intermittent rearrangements, rather than a continuous process. Past investigations on 2D gel-forming Langmuir monolayers^50^, coarsening processes in colloids undergoing spinodal decomposition^21^, aged laponite^31^ and metallic glasses^51^ found a partial agreement with this latter model. In the particular case of 2D gels, this phenomenology was present, even with a larger variation of *γ* than theoretically predicted. From that, a nice master curve could be deduced, from which the typical length of the jump could be extracted.

The present case seems to be quite different, as the strong dependence of *γ* on 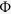 is not accompanied by any detectable Q-dependence, as shown in Fig. S3 of Supplementary Information. The reason for the difference is could be either related to the insufficiently large Q-range explored (which however seems unlikely, given that it is not any smaller than that explored in other XPCS experiments) or rather being related to different physical mechanism underlying dynamical arrest in the present case.

Since the mechanical moduli of this mixed monolayer are somewhat smaller than those of the pure DPPC monolayer at the same pressure, it seems reasonable to assume that the disks behave as rigid bodies (as they are formed by pure DPPC in LC phase) which repel each other and are surrounded by a softer phase. Then the system would be more similar to a repulsive glass than to a gel-forming system, as described by the aforementioned models.

In this respect, we note that the dilational elasticity 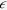 increase with 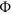 following a power law trend, 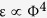, as shown in Fig. S6 in Supplementary Information. The trend is compatible with the increase of the relaxation time 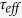 with 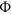 shown in Fig. 6a. This dependence is much weaker than the 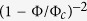 trend found for hard disks close to the critical concentration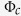, and weaker than the 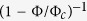 dependence due to long range electrostatic potentials, found e.g. in tri-components phospholipid layers showing phase coexistence^52^. The much weaker power-law trend found in our system is likely to be connected with the presence of the softer, mixed NP-DPPC phase surrounding LC DPPC domains, as e.g. in systems composed of core-shell PNIPAM spheres^53^.

Finally, one could ask whether the nanoparticle size plays an important role in this complex phenomenology. We tested different sizes of NPs, namely 9 nm, 15 nm and 60 nm particles. As the results displayed in Fig. 6 demonstrate, the nanoparticle size plays no important role in this phenomenology, in the same way, as the nanoparticle size is not affecting the structure of the system (Fig. 3).

## Conclusions

Through the combined use of GI-XPCS and epifluorescence microscopy measurements (MT and DFM), we have been able to characterize the dynamical regimes of a hybrid, self-organized phospholipid/NP monolayer observed while increasing its surface pressure and concentration.

**Figure 8:**
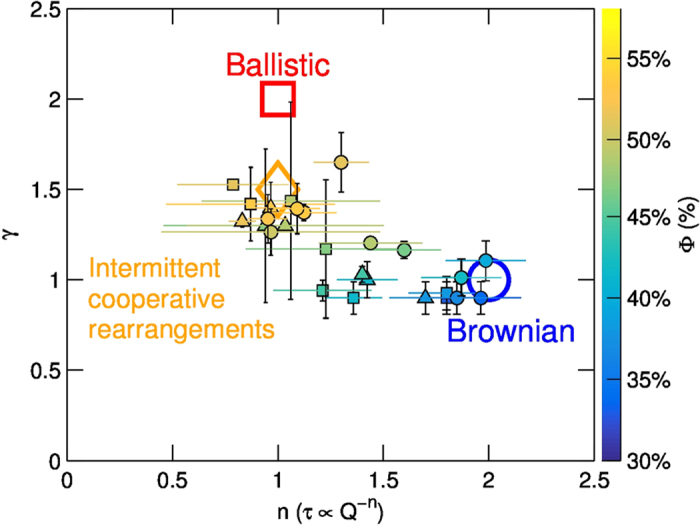
The different dynamical regimes discussed in the paper, Ballistic regime, Brownian diffusion and Intermittent Cooperative Rearrangements occupy different region in the space of parameters. The data measured by XPCS (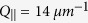, the same as in Fig. 5) are reported as filled symbols. The color of each point is chosen to represent the corresponding value of 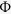 at which it has been measured.

Silica NPs are incorporated into the film: a stable NP/DPPC mixed matrix is formed, that surrounds regions of tightly packed DPPC molecules in LC domains. Those form rigid disks, which retain their own individuality and cage each other as the surface is compressed. As the packing fraction of the disks increases, we observed a dynamical transition from Brownian diffusion to an arrested dynamical regime, featuring intermittent, cooperative rearrangements in a fashion closely resembling that found in simulations^54^ and experimental characterizations^55^,^56^ of 3D colloidal supercooled fluids approaching the critical point. These results indicate that a residual dynamics remains present even in the densely packed structure formed. To summarize the parameters describing the dynamical transition observed, we report the XPCS results in Fig. 8 as a function of 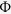 on a 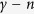 plane; the color of each point corresponds to its corresponding surface concentration.

The observed peculiar structure of the mixed particle-DPPC monolayer- and its dynamic characteristics- can be used to explain some of the observations already reported on the chemico-physical features of these monolayers. In particular, in all range of investigated surface pressures, the rearrangement and relaxation of the monolayer structure is dramatically affected by the presence of NPs, which explains the increased non linearity of the surface pressure response to area oscillatory cycles and its large hysteresis on compression/expansion cycles.

The reported results can also be important in relation to possible effects of NPs on the dynamics of cell membrane (membrane fluidity, rafts), which have been so far poorly investigated.

More generally, we want to stress here the importance of using a comprehensive suite of experimental tools to characterize the slow dynamics of soft and bio-systems, often strongly deviating from a simple Brownian behavior. This is particularly true in view of possible applications that exploit slow, relaxation mechanisms to extend performances and enhance key features e.g. in the fields of drug release and of self-healing materials. The partial overlap of spatial scales explored by MT, DFM and GI-XPCS allowed us to successfully combine them to gain a deeper insight into the dynamics of the system, exploiting the strong points of each technique while extending the spatial range probed: by microscopy we tracked the dynamics on lengths from 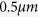 up to 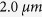, while with this particular GI-XPCS experiment we probed the system on scales ranging from 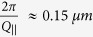 up to 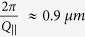. On other systems, MT and GI-XPCS could in principle access larger ranges, corresponding to the colored areas in Fig. 7.

## Materials and Methods

The NP suspensions used here are obtained by dilution with pure water of different commercial colloidal dispersions products: Levasil 300/30, Levasil 200/30 and Levasil 50/50, all from H.C. Starck (Germany). The NPs in the three dispersions are spherical and narrowly distributed around diameters of 9, 15 and 60 nm, respectively. The dispersions are extremely stable because of the large negative surface charge of the particles, obtained in the specific production process. The NP 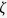-potential is in fact 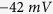 and the dispersions pH is around 9. Thus, the dispersions do not contain any stabilizing agent that could interfere with the studied properties. This is also confirmed by the measurement of the surface tension, which provides for all dispersions values similar to those of pure water: about 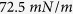, stable for several hours.

DPPC (MW 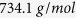, purity 99%) is supplied by Sigma (Germany) and used without further purification. Pure water is produced by a two stage system (Millipore, Elix plus Milli-Q) providing a resistivity greater than 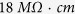 and a surface tension of 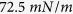 without any appreciable kinetics over several hours. For the micro epi-fluorescence measurements, a small quantity of the fluorescence probe NBD-PC, (Avanti Lipids) was added to the spreading solution of DPPC (1% wt of the total lipid content).

### Langmuir Monolayer Preparation

A Langmuir trough, cleaned with organic solvents, is filled with a suspension of colloidal particles (1% wt concentration). The subphase temperature is controlled by means of water circulation between the trough basement and a LAUDA thermostat, set at 20 °C.

Small constant velocity drifts of the Langmuir film are inevitable: in order to minimize them, a steel ring (1 cm diameter) is placed in the subphase, centered under the measurement region. There, it reduces the subphase depth around the measurement region, effectively minimizing subphase motions. A chloroform solution of DPPC is spread at the interface between air and the aqueous suspension of NP. The trough is covered with a plastic cap to avoid dust deposition and an incubation time of 1 hour is considered: the process of formation of the composite monolayer needs the adsorption of NP at the interface, which is presumably a slow equilibration between NPs in subphase and at the interface.

Subsequently, the trough’s area is reduced by means of the barrier motion, to reach the desired surface pressure value. Barriers have been halted before starting any measurement. All the techniques employed (MT, DFM, XPCS) act on a much shorter time scale than that of the equilibration process between surface and bulk. Moreover, once the structures of Fig. 3 are formed by compression of the monolayer, they are found to be stable even for several hours.

### Epifluorescence and Microscopic Tracking

A close insight into the properties of the mixed layer has been obtained by fluorescence microscopy analysis. Measurements were performed using a Nikon Ti-Eclipse inverted microscope equipped with a high-sensitivity Andor Clara camera.

To track the dynamics, in correspondence of several surface pressure points on the Langmuir isotherm, videos have been recorded with 50× magnification (Nikon LU Plan EWLD 50×/0.55B, 1 pixel = 0.36 microns), at 7 frames per seconds, with exposure time of 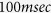. Every video has been analysed using ad hoc developed Matlab code in order to extract the mean square displacement (MSD) *x*^2^ of the objects at a given surface pressure. We take advantage of the large number of objects (N > 100) in the field of view to subtract the film drift motion by calculating the MSD from the variation of the inter-object distances over time:


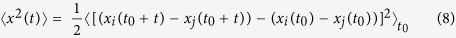


The average is performed along the statistical ensemble of inter-object distances in the field of view. Moreover, each trajectory has been divided into smaller portion that were subsequently included in the average, in order to improve the accuracy of 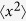 at short times. Mean square displacements have been corrected for static tracking errors following the procedure of Savin and Doyle^57^. Strongly non-circular features (eccentricity 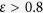) have not been considered in the analysis, because of the less precise position determination connected to non-circular shapes. Less than 2% of the objects have been discarded. More details on the tracking algorithm and on the measurement of diffusion coefficients are available in the Supplementary Information.

### Grazing Incidence X-ray Photon Correlation Spectroscopy

Photon correlation spectroscopy experiments have been performed in grazing-incidence, small angle scattering geometry (GI-XPCS) using partially coherent X-rays at the ID10 beamline of the European Synchrotron Radiation Facility. A Si (111) crystal monochromator is used to select radiation of wavelength 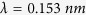. The transversely coherent beam is defined by slit blades with highly polished cylindrical edges; the parasitic scattering due to diffraction from these slits is blocked by a set of guard slits. The beam is directed to impinge at grazing incidence (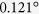, i.e. 80% of the critical angle for total reflection) on the Langmuir trough. The scattered radiation travels in vacuum to minimize parasitic scattering and in-air absorption, reaching a Medipix detector placed 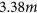 downstream the sample where it is collected. A motorized metallic finger blocks the reflected beam.

Speckle patterns are recorded with exposure times in the range 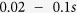. The experiments covered a 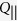 range from 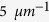 to 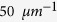 with 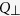 ranging from 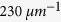 to 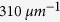.

As usual, in the case of Langmuir monolayers^45^,^50^ the dynamics are shown to depend only on the parallel component of the scattering vector; no dependence on the perpendicular component has been found (see Fig. S2 in the Supplementary Information). In view of this, all the ensuing analysis has been performed averaging the scattered intensity along the 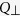 direction, in order to improve the signal-to-noise-ratio. From the scattered intensity measured by each group of pixels as a function of time, we calculate the intensity autocorrelation function


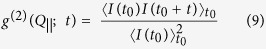


using a fast and efficient multi-tau algorithm coded in Matlab.

### Digital Fourier Microscopy

Information on the microscopic dynamics of the sample can be re-cast in the momentum space: from the very same epifluorescence images used for microscopic tracking we calculated the power spectrum of the 2D Fourier transform of each image of a given sequence. Then, each pixel of the transformed image corresponds to a well-defined momentum vector Q. Pixels are grouped in rings, in the range 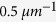 to 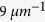, each being labelled by the corresponding modulus |Q|. Then, autocorrelation functions of the power spectrum have been calculated using the same multi-tau algorithm used for XPCS. These functions are formally analogous to those obtained by XPCS.

## Additional Information

**How to cite this article**: Orsi, D. *et al*. 2D dynamical arrest transition in a mixed nanoparticle-phospholipid layer studied in real and momentum spaces. *Sci. Rep*. **5**, 17930; doi: 10.1038/srep17930 (2015).

## Supplementary Material

Supplementary Information

## Figures and Tables

**Figure 1:**
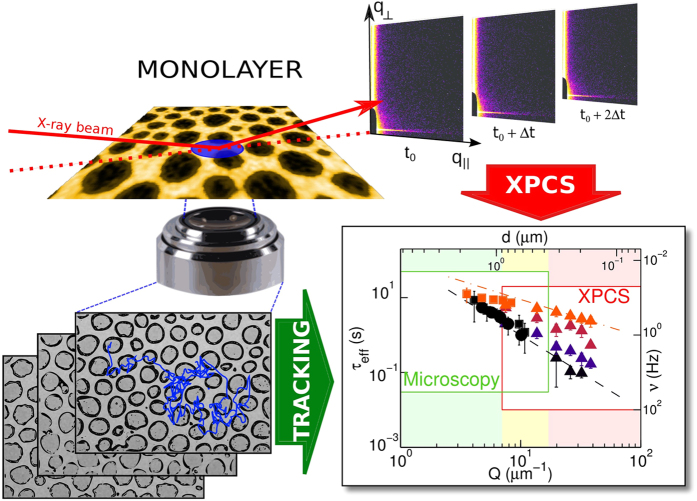
The combination of grazing-incidence x-ray photon correlation spectroscopy and epifluorescence microscopy provides both excellent statistics and unambiguous determination of the dynamical model covering a much larger Q-range than any single technique.
