# Supplementary material for: 2D dynamical arrest transition in a mixed nanoparticle-phospholipid layer studied in real and momentum spaces
Source: Sci Rep. 2015 Dec 10;5:17930. doi: 10.1038/srep17930 (PMC4674700; doi:10.1038/srep17930)
Supplement: Supplementary Information [file srep17930-s1.pdf]

2D dynamical arrest transition in a mixed nanoparticle-phospholipid layer studied in real and momentum spaces

Davide Orsi[1*], Eduardo Guzmán[2], Libero Liggieri[2], Francesca Ravera[2] ,Beatrice Ruta[3] , Yuriy Chushkin[3], Tiziano Rimoldi[1], Luigi Cristofolini[1*]

1- Dipartimento di Fisica e Scienze della Terra, Università degli Studi di Parma, Parma, Italy.

2- Consiglio Nazionale delle Ricerche - Istituto per l'Energetica e le Interfasi, U.O.S. Genova (CNR IENI), Genova (Italy).

3- ESRF- The European Synchrotron, CS 40220, 38043 Grenoble Cedex 9, France

SUPPLEMENTARY INFORMATION:

Details of the microscopic tracking algorithm.

To track the dynamics, in correspondence of several surface pressure points on the Langmuir isotherm, we recorded videos at the epifluorescence microscope using a 50x objective (Nikon LU Plan EWLD 50x/0.55B) at 7 frames per seconds. Each frame was taken with exposure time $t_{exp} = 0.1sec$. We used a Nikon Eclipse Ti inverted microscope, controlled using the MicroManager Software, version 1.4 [1], that was equipped with a custom-built Langmuir trough placed on the microscope table. The microscope is equipped with an Andor Clara high sensitivity camera: its sensor counts 1392×1040 pixels, each $6.45\mu m \times 6.45\mu m$ in size. Images sequences have been saved as 16bit, uncompressed tiff images. In order to improve the fluorescence intensity, we applied a 2X2 binning to the images; this resulted in pixel/length conversion factor of 0.36 microns per pixel.

Image analysis was performed using our software developed in the Matlab computing environment (Mathworks Inc). In the following, we report details on how the tracking algorithm works. Raw epifluorescence images have been

1- Filtered with a Gaussian filter (standard deviation = 3 pixels),

2- Inverted, in order to have white features on black background,

3- Converted to binary images, applying an adaptive threshold algorithm– an algorithm that applies different threshold value in different regions of the image – in order to account for non-planar background.

The eccentricity of the features has been measured and used to discard non-circular objects. The bounding box of each object is retrieved from the binary image: it is used to select the pixels that compose the object in the raw image. The grayscale intensity is then used to determine the position of the diffusor, by means of parabolic fitting around the maximum of the intensity.

This algorithm is analogous with the one from Crocker and Grier[2] with respect to the functions that label each moving feature in the frames, linking their position into trajectories; the algorithm was optimized for performance using Matlab routines.

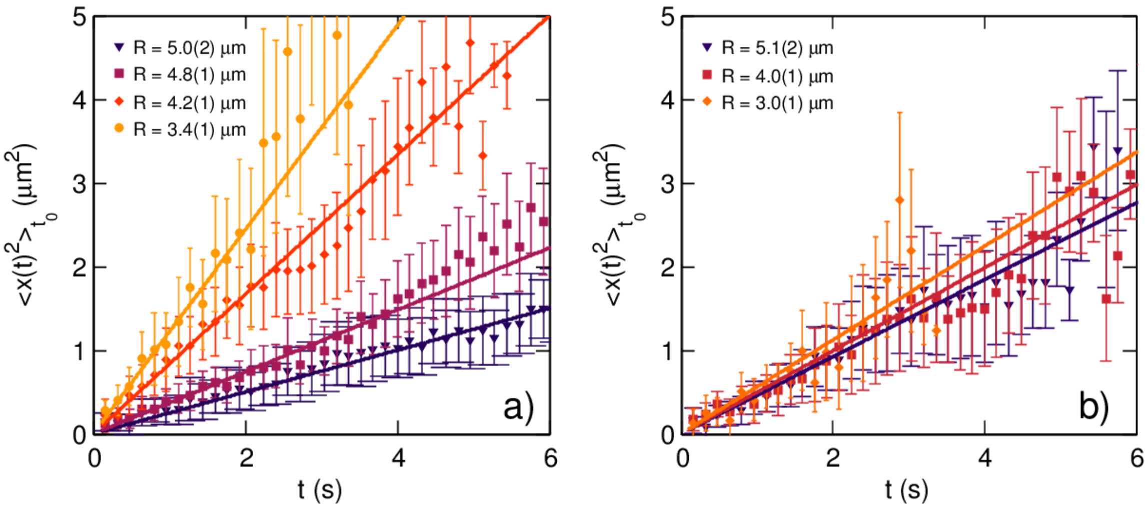

Figure S1*: mean square displacements of domains with different radius, measured at a) $\Phi = 30.6\%$ and b) $\Phi = 52.4\%$. In (a), low Φ, the slope (proportional to the diffusion coefficient) is proportional to 1/R, while in (b), high Φ, it shows negligible R-dependence.*

Figure S1 reports the time-averaged mean square displacements of a selection of individual objects with different radius, measured at $\Phi = 30.6\%$ (figure S1-a) and at $\Phi = 52.4\%$ (figure S1-b). While in the former case we find that the diffusion coefficient is proportional to 1/R, in the latter no dependence on the radius is found. This behavior has been summarized in Figure 3 of the article and it is discussed in the text.

In order to improve the accuracy of the analysis of the diffusion coefficients reported in Figure 3 of the article, features have been grouped in ensembles, depending on their radius; the width of each ensemble is $0.15\mu m$. For each ensemble, an averaged MSD is calculated.

Details of the GI-XPCS experiments.

This paragraph reports additional information on the calculation and fitting of the intensity autocorrelation functions in XPCS experiments. The pixels of the 2D detector have been grouped into square regions of adjacent pixels, each group labeled by the corresponding scattering vector components $(Q_{||}, Q_{\perp})$. We calculate the autocorrelation function of the scattered intensity measured by each group of pixels as a function of time,

$$g^{(2)}(Q_{||}, Q_{\perp};\, t) = \frac{\langle I(t_0) I(t_0+t)\rangle_{t_0}}{\langle I(t_0)\rangle_{t_0}^2}, \qquad \text{[S1]}$$

using a multi-tau algorithm developed at the ID10 beamline, ESRF. Correlation functions are compared with the Kohlrausch-William-Watts (KWW) exponential decay

$$g^{(2)}(q_{||}, q_{\perp};\, t) = A + \beta e^{-2\left(\frac{t}{\tau}\right)^{\gamma}}, \qquad \text{[S2]}$$

where τ is the relaxation time of the dynamics, β is the contrast and γ, called "shape parameter", indicates the character of the microscopic dynamics.

We observe no dependence of the fitting parameters on the component of the scattering vector perpendicular to the air/water interface $Q_\perp$. To highlight this, we report in figure S2-a a color plot that represents the relaxation time τ as a function of $(Q_{||}, Q_\perp)$; the absence of a $Q_\perp$ dependence is evident. As a consequence of this, in all the subsequent analysis we improved the signal to noise ratio of the correlation functions by integrating over $Q_\perp$. An example of the resulting averaged correlation functions are reported in figure S2-b, for several values of $Q_\parallel$; continuous lines are fit to equation [S2].

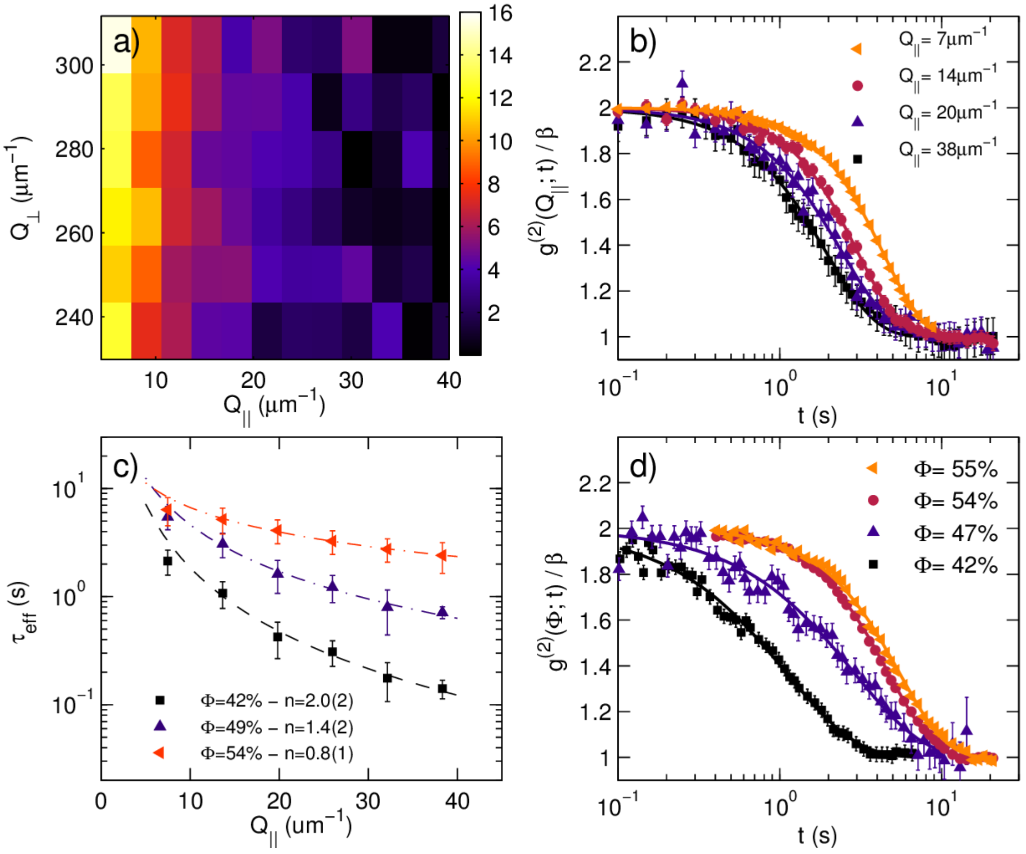

Figure S2: *a) the relaxation time is proportional to the inverse of* $Q_\parallel$*, the component of the scattering vector parallel to the water surface; no dependence along the perpendicular component* $Q_\perp$ *is found. Panel shows data relative to the DPPC/120nm NPs sample at* $\Phi = 55\%$*. b) Correlation functions measured at several values of* $Q_\parallel$ *and* $\Phi = 55\%$*. Lines are fits to equation S2. c) Details of the* $\tau(Q_\parallel)$ *dependence at selected values of* Φ*. Continuous lines are fit to* $\tau \propto Q_\parallel^{-n}$ *d) The shape of the correlation function changes with increasing* Φ*, from simple to compressed exponential; at the same time, relaxation time increases. The panel reports normalized correlation functions measured at different values of* Φ*, at* $Q_\parallel = 6\mu m^{-1}$*. Continuous lines are fit to equation S2.*

Panels c and d of figure S2 detail the surface concentration dependence of the correlation functions. Panel c shows the $Q_\parallel$ dependence of the relaxation time for selected values of Φ; lines are fits with

power-law decays $\tau \propto Q_{\|}^{-n}$. The panel highlights the change of the power law exponent from $n{\sim}2$ to $n{\sim}1$ as Φ increases, which is summarized in figure 5 of the article. Panel d shows correlation functions measured at fixed $Q_{\|} = 6\mu m^{-1}$, in correspondence of different values of Φ; aside from the increase of τ, the most evident feature is the change of their shape as Φ grows, from simple exponential decays to faster-than-exponential (compressed) decays.

The observed evolution of the two parameters n and γ marks the dynamical transition from Brownian motion to the arrested dynamics characterized by intermittent rearrangements.

The shape parameter γ is found to be constant over the Q-range probed, as shown by the fact the the correlation function measured at different values of $Q_{\|}$ and reported in figure S2b all decay following the same trend. In figure S3 we report the Q-dependence of γ measured at $\Phi = 53\%$. Each point is obtained as the average value over four different correlation functions measured at the same value of Q in different locations of the sample; error bars are standard deviations. The plot shows that γ is constant in the Q-range probed; analogous results are found at all the surface concentrations investigated.

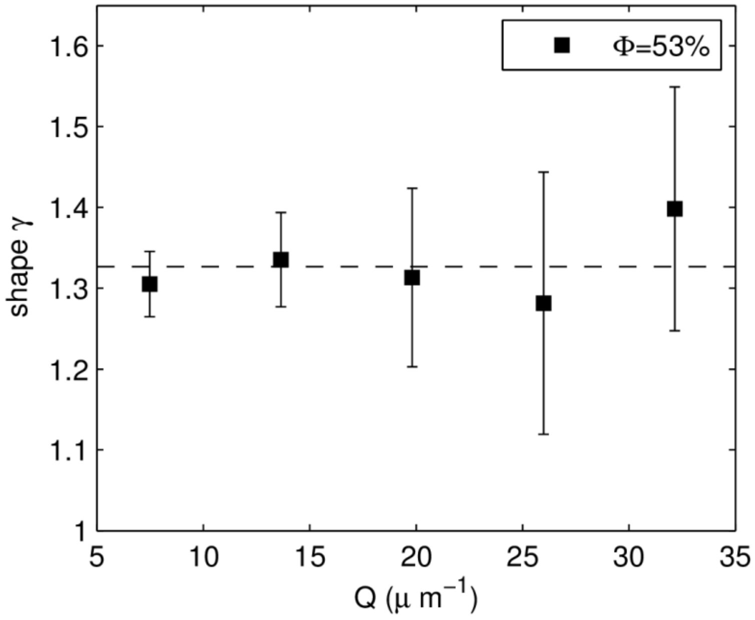

***Figure S3**: The shape parameter γ is constant over the Q-range probed. The plot reports the average value over four different correlation functions measured at the same value of Q in different locations of the sample, at $\Phi = 53\%$; error bars are standard deviations.*

Details of the Discrete Fourier Microscopy

Figure S4 reports an example of DFM analysis on images recorded at $\Phi = 52\%$. In panel a) we report an epifluorescence micro-image, in panel b) its Fourier transform, obtained by the fft2 algorithm within Matlab®; concentric black circles separate rings having different modulus of momentum |Q|, in the range from $4\ \mu m^{-1}$ to $9\ \mu m^{-1}$.

Vertical and horizontal black stripes mask some unavoidable artifacts that are due to the finite size of the image to be transformed. Panel c) reports the correlation functions obtained in this way, together with their best fit with a Kohlrausch-William-Watts law (eq. [5]).

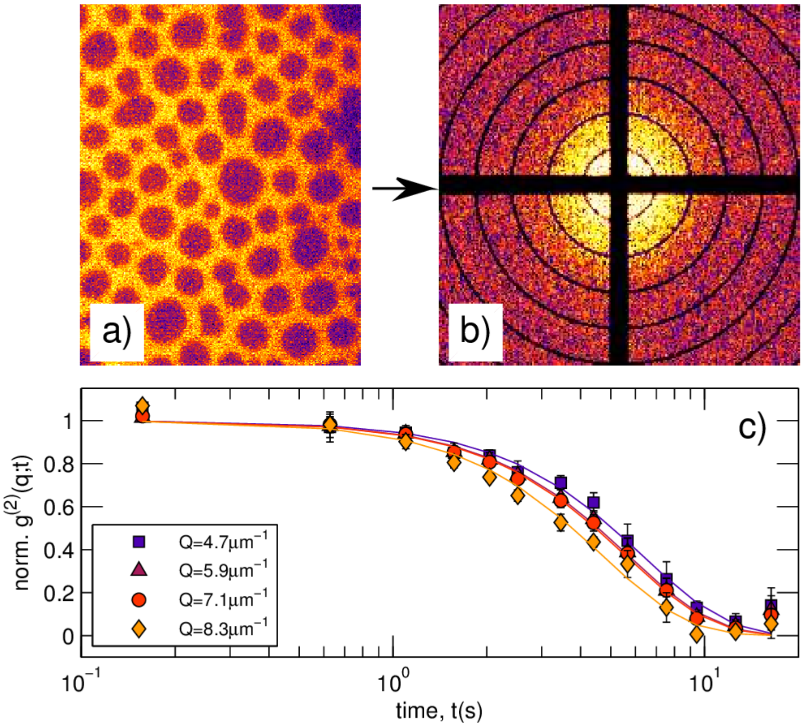

***Figure S4**: the epifluorescence images (a) are transformed into the Q-space using a FFT algorithm (b). The autocorrelation functions of the Fourier power spectrum decay as KWW exponentials (eq.5): panel c) reports in a plot the DFM autocorrelation functions obtained in this way; lines are fits with eq.5.*

To investigate the presence of intermittent rearrangements in the sample, we analyzed the fluctuation of the Fourier power spectrum of the difference of two consecutive images. Figure 5d in the paper reports the histogram of the fluctuations of this signal, with respect to its time-averaged value. Here in figure S5 we report the fluctuations as a function of time.

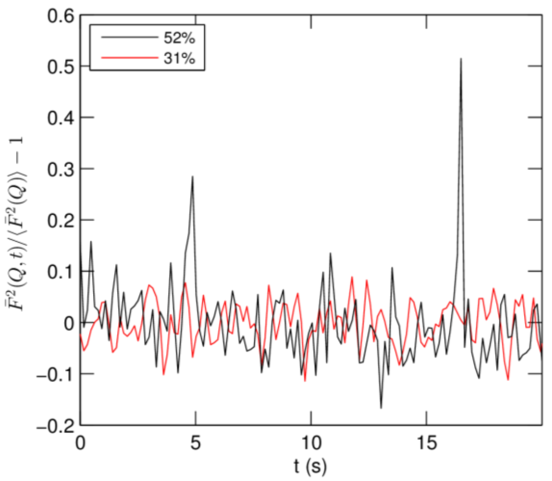

Figure S5*: temporal fluctuations of the Fourier transform power spectrum, with respect to its average value, measured t* $Q = 3.5\mu m^{-1}$ *for two different values of surface concentration* $\Phi = 31\%, 52\%$. *Large fluctuations, indicative of fast rearrangements, are found at* $\Phi = 52\%$.

Mechanical Properties of the Langmuir Monolayer

The quasi-static dilational modulus ε of the DPPC-NP monolayer was measured by applying a sinusoidal variation to the area available to the film (typically $^{\delta A}/_{A} \sim 2 - 3\%$) and simultaneously measuring the variation in the surface pressure Π. The results are shown in Figure S6.

The increase of XPCS relaxation time τ and of ε follow the same dependence from the concentration Φ. At a phenomenological level, this can be described as the power law $\varepsilon \propto \Phi^4$. This dependence is much weaker than the $\left(1 - {^{\Phi}}/{_{\Phi_c}}\right)^{-2}$ regime connected to a hard disks repulsive potential in which the modulus diverges at the critical concentration Φ_c. In monolayers of phospholipids (DPPC/POPG) mixed with palmitic acid or n-hexadecanol, which exhibit LC/LE phase-coexistence, Ding and co-workers[3] found that the viscosity increases following a $\left(1 - {^{\Phi}}/{_{\Phi_c}}\right)^{-1}$ law due to a long range electrostatic potential between phospholipid LC domains. The much weaker dependence found in our system may be connected to the presence of NPs in the mixed phase, whose electrostatic charge may screen the long range electrostatic potential. This is also supported by the fact that similar power law trends have been found in many core-shell 3D colloidal systems, e.g. Koumakis[4] and coworkers found a Φ^7 dependence in soft PNIPAM spheres. In our system, the mixed DPPC/NP matrix may act as an outer shell layer, resulting in an effective 2D core-shell system. It would be interesting to compare this phenomenology with other 2D core-shell systems.

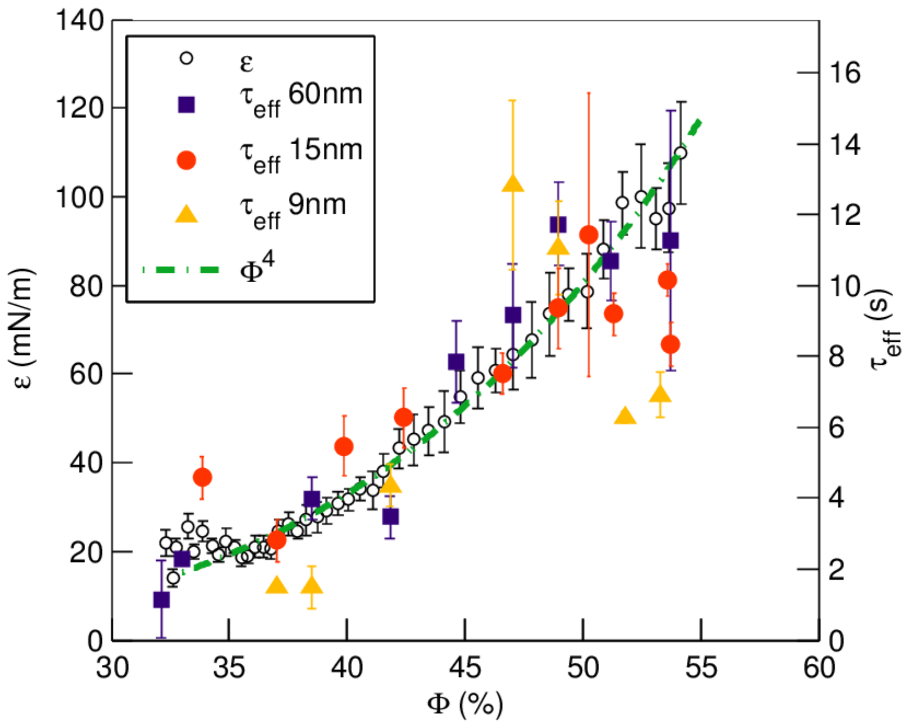

Figure S6: *Both the quasistatic dilational modulus ε (open circles, left axis) and the XPCS relaxation times τ (colored) increase with Φ. The dashed line is a fit to a Φ^4 dependence.*

1. Edelstein, A., Amodaj, N., Hoover, K., Vale, R. & Stuurman, N. Computer control of microscopes using µManager. *Curr. Protoc. Mol. Biol.* **Chapter 14,** Unit14.20 (2010).

2. Crocker, J. C. & Grier, D. G. Methods of Digital Video Microscopy for Colloidal Studies. *J. Colloid Interface Sci.* **179,** 298–310 (1996).

3. Ding, J., Warriner, H. & Zasadzinski, J. Viscosity of two-dimensional suspensions. *Phys. Rev. Lett.* 16–19 (2002). doi:10.1103/PhysRevLett.88.168102

4. Koumakis, N., Pamvouxoglou, a., Poulos, a. S. & Petekidis, G. Direct comparison of the rheology of model hard and soft particle glasses. *Soft Matter* **8,** 4271 (2012).
